# Modelling the 5‐Year Impact of Twice‐Yearly Injectable Lenacapavir for HIV Pre‐Exposure Prophylaxis in the United States

**DOI:** 10.1002/jia2.70188

**Published:** 2026-08-10

**Authors:** Mia Moore, Serin Lee, Laura Matrajt, Sarah E. Stansfield, Li Tao, Juan Yang, Dylan Mezzio, James Jarrett, JeanPierre Coaquira Castro, Alice Hsiao, Woodie Zachry, Dobromir Dimitrov

**Affiliations:** ^1^ Vaccine and Infectious Disease Division Fred Hutchinson Cancer Center Seattle Washington USA; ^2^ Department of Industrial and Management Engineering Incheon National University Incheon South Korea; ^3^ Department of Applied Mathematics University of Washington Seattle Washington USA; ^4^ Gilead Sciences, Inc. Foster City California USA

**Keywords:** epidemiological models, HIV‐1, pre‐exposure prophylaxis, sexual and gender minorities, statistical models, United States

## Abstract

**Introduction:**

The impact of pre‐exposure prophylaxis (PrEP) on the US HIV‐1 epidemic has not been fully maximized, potentially due to challenges in uptake, coverage, adherence and consistent use. Following the FDA (Food and Drug Adminstration) approval of twice‐yearly lenacapavir, epidemiological projections are needed to understand the potential role of lenacapavir in overcoming PrEP barriers. The objective of this analysis was to estimate the impact of lenacapavir on HIV‐1 incidence in the United States.

**Methods:**

We used a static cohort model representing the US population aged 15 or older to predict the number of HIV‐1 cases averted between 2026 and 2030 under different lenacapavir uptake scenarios (S1–S5) versus projected status quo 2025 PrEP use with no lenacapavir (S0). In S0 and S3, we assumed 2025 levels of PrEP use (652K total PrEP users with no lenacapavir use) for 2026–2030. In all other scenarios, we assumed PrEP use increases to 1.1 million PrEP users with 0%−100% using lenacapavir. Oral and injectable PrEP effectiveness were based on clinical trials adjusted by percent days covered in real‐world use. Oral PrEP use was assumed to be zero among people who inject drugs in all scenarios.

**Results:**

In S1, moderate lenacapavir uptake (37% of PrEP users by 2030) averted 13,700 (95% credible interval: 10,800–18,300) new HIV‐1 cases, an 8% (6%–10%) reduction in cumulative cases between 2026 and 2030 relative to S0. In S2, more rapid lenacapavir uptake (85% of PrEP users by 2030) averted 23,800 (19,100–33,700) cases. Switching all PrEP users to lenacapavir averted 22,400 (12,200–47,200) and 37,400 (26,800–61,200) cases without (S3) and with (S4) an increase in PrEP use, respectively. In S5, 9400 (4600–11,500) cases were averted if PrEP use increased but lenacapavir was not introduced. This analysis was limited as only reductions in primary acquisitions were considered in the static cohort model and the model did not account for potential future demographic or behavioural shifts.

**Conclusions:**

Twice‐yearly lenacapavir may impact the US HIV‐1 epidemic by improving adherence and increasing the duration of protection relative to oral PrEP, leading to more consistent protection over time and reducing HIV‐1 incidence.

**Abbreviations**
ACS: American Community Survey
F/TAF: emtricitabine/tenofovir alafenamide
F/TDF: emtricitabine/tenofovr disoproxil fumarate
MCMC: Markov Chain Monte Carlo
MSM: men who have sex with men
NHSS: National HIV Surveillance Study
PDC: proportion of days covered
PrEP: pre‐exposure prophylaxis

## 1 Introduction

Pre‐exposure prophylaxis (PrEP) for prevention of HIV‐1 acquisition was approved by the US Food and Drug Administration in 2012, with the introduction of daily oral emtricitabine/tenofovir disoproxil fumarate (F/TDF) [1]. Subsequently, daily oral emtricitabine/tenofovir alafenamide (F/TAF) and cabotegravir, an every‐2‐month intramuscular injection, were approved in 2019 and 2021, respectively [2, 3]. More recently, the twice‐yearly subcutaneous injection, lenacapavir, was approved in June 2025, based on the results from two Phase 3 studies, PURPOSE 1 (NCT04994509) and PURPOSE 2 (NCT04925752) [4, 5, 6]. Both studies demonstrated superiority for twice‐yearly lenacapavir over F/TDF for the prevention of HIV‐1 acquisition [4, 5].

Although PrEP prescriptions have steadily increased since initial approval [7], in 2023, there were 13.6 new HIV‐1 diagnoses per 100K people in the United States, the highest number since 2017 and only marginally fewer than the 13.9 per 100K reported in 2017 [8]. New HIV‐1 acquisitions disproportionately affect specific populations such as men and transgender women who have sex with men (MSM/TGW) and people who inject drugs (PWID), widening health disparities across ethnicities, genders, geographies and races, and generating a lifetime burden of HIV‐1 [9]. Furthermore, the average annual and cumulative healthcare costs are up to seven times higher for people living with HIV‐1 compared with those without HIV‐1 [10]. Daily oral PrEP is highly efficacious with consistent use [11]; however, adherence and persistence to daily oral PrEP decline significantly during the first 6 months of use, decreasing its effectiveness in both clinical trial and real‐world settings [12]. High population‐level effectiveness requires PrEP uptake (i.e. initiation) and persistence within communities concordant with their HIV‐1 exposure; however, PrEP uptake relative to need is lower among Black and Hispanic individuals, and PrEP persistence is lower among younger individuals, despite greater HIV‐1 incidence [13, 14]. Despite these challenges, US states with higher PrEP coverage saw a greater reduction in new HIV‐1 diagnoses per capita from 2012 to 2022 [7].

Injectable PrEP options have the potential to increase the population‐level impact of PrEP in three ways. First, clinical trials of active PrEP users have demonstrated the superiority of every‐2‐month cabotegravir and twice‐yearly lenacapavir over F/TDF for the prevention of HIV acquisition [4, 5, 15, 16]. Second, longer‐acting injectables may be less impacted by PrEP discontinuation, due to the longer intervals between administration. In particular, a single lenacapavir injection provides at least 6 months of protection, even if the individual discontinues PrEP [14]. Third, injectable PrEP regimens may increase PrEP uptake, as potential and current PrEP users express a preference for a range of prevention options [17, 18].

Mathematical model‐based projections of the impact of cabotegravir have demonstrated the importance of increased uptake and persistence, though not necessarily increased clinical effectiveness (efficacy + adherence) [19, 20, 21, 22]. A study of 32 cities in the United States predicted that cabotegravir could reduce HIV‐1 incidence by 36% in MSM by 2030 with 10% additional PrEP uptake, but only 4% with no additional PrEP uptake [19]. Similarly, a model of MSM in Atlanta, Georgia, predicted that doubling PrEP coverage, with 50% of all PrEP users receiving cabotegravir, would avert 17% of new HIV‐1 cases over 10 years, whereas switching 50% of users to cabotegravir with no overall increase in coverage would avert only 4% of cases [20]. Finally, a model comparison of cabotegravir expansion over 20 years in three high‐resource settings found that PrEP coverage was a stronger driver of averted new HIV‐1 cases than choice of product [21].

Quantitative projections of the population‐level impact of lenacapavir can inform policies to ensure access and maximize its potential to reduce new HIV diagnoses. In the present study, a static cohort model was used to quantify the potential impact of lenacapavir by projecting the number of new HIV‐1 cases averted over 5 years across the entire US population from 2026 to 2030, relative to a baseline of status quo PrEP use. These projections were based on the following: the demonstrated superiority of lenacapavir relative to F/TDF in the PURPOSE 1 and PURPOSE 2 studies; detailed data on trends in PrEP uptake, persistence and adherence, and product choice by different subpopulations; and a set of potential scenarios for how lenacapavir might be used based on those trends. A 5‐year time horizon was chosen to (1) focus on the impact of PrEP on primary infection rather than onward transmission, which is not accounted for in a static cohort model, and (2) allow for significant changes in PrEP use following current trends.

## 2. Methods

2

### 2.1 Modelled Population

**FIGURE 1:**
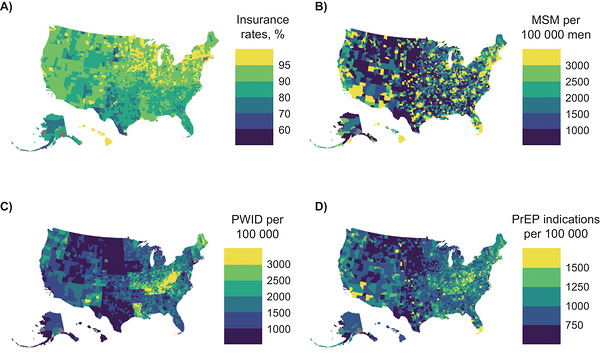
Population structure used in PrEP simulations by (A) insurance coverage, (B) distribution of MSM, (C) distribution of PWID and (D) PrEP indications. In (A), insurance coverage rates were taken from the US Census and include both public and private insurance. In (B), the distribution of MSM was based on American Community Survey data. In (C), the distribution of PWID was derived from overdose deaths. In (D), PrEP indications were based on estimated numbers of MSM and PWID, with heterosexuals assumed to be uniformly distributed. Abbreviations: MSM, men who have sex with men; PrEP, pre‐exposure prophylaxis; PWID, people who inject drugs.

A static cohort model representing the entire US population aged 15 years or older was developed and stratified by county, age, race, gender, sexual behaviour, insurance status and injection drug use (Figure 1; full details in the Supplementary Methods and Tables S1 and S2). The number of MSM by county was estimated using data from the American Community Survey (ACS) [23]. The rate of injection drug use was estimated by county and demographic variables based on frequency of fatal drug overdoses [24]. Insurance coverage, defined as any public or private insurance, was stratified by age, race and county using ACS data [25].

### 2.2 PrEP Scenarios

A detailed model of future PrEP use was developed through 2030, stratified by county, race, age, gender and insurance status; full details in the Supplementary Methods, Tables S2–S4, and Figures S1 and S2. The growth rate of the overall number of PrEP prescriptions was calibrated to National HIV Surveillance Survey (NHSS) data from 2012 to 2023. PrEP uptake, proportion of days covered (PDC) and product choice (F/TDF, F/TAF, cabotegravir or a mixture of F/TDF and F/TAF) were calibrated to national‐level data from 2021 to 2024 from a commercial data set, IQVIA Real‐World Longitudinal Prescriptions and Pre‐Adjudicated Claims. PDC was calculated as the percentage of days during the 1‐year period beginning on the date an individual first received PrEP for which there was a sufficient supply of any prescribed PrEP regimen to provide protection based on refills (for oral PrEP) or injection dates (for injectable PrEP). Because PDC was calculated as a population‐level measure, it is not restricted to the discrete values corresponding to individual injections.

**FIGURE 2:**
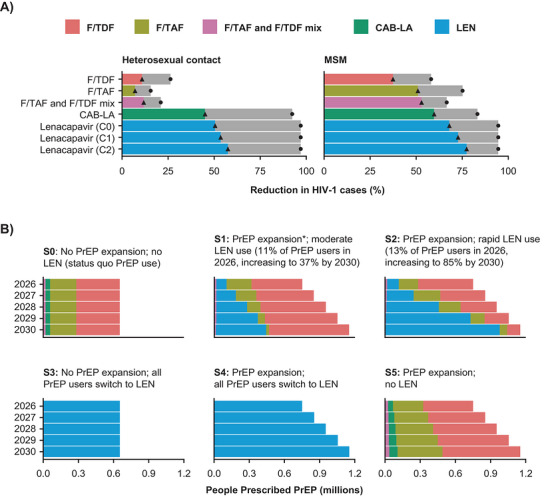
Overview of model assumptions for (A) PrEP effectiveness and (B) number of individuals prescribed PrEP in each year under different scenarios S0–S5. *The percentage of generic F/TDF users remained stable over time. In (A), PrEP effectiveness was defined from clinical trials (intention‐to‐treat population; indicated by circles) and real‐world PrEP effectiveness defined by PDC (triangles). In (B), six different PrEP uptake scenarios are shown. In S0 and S3, the model assumes 2025 levels of PrEP users for 2026–2030 without any additional uptake; in S1, S2, S4 and S5, the model assumes an increase in the number of PrEP users from 652,000 in 2025 to 1,150,000 in 2030. Colours representing different PrEP products are consistent across (A) and (B). Abbreviations: CAB‐LA, long‐acting cabotegravir; F/TAF, emtricitabine/tenofovir alafenamide; F/TDF, emtricitabine/tenofovir disoproxil fumarate; LEN, lenacapavir; MSM, men who have sex with men; PDC, proportion of days covered; PrEP, pre‐exposure prophylaxis.

The PDC for lenacapavir in real‐world settings remains unknown; therefore, three scenarios were considered labelled C0, C1 and C2 (Table 1). In scenario C0, the base case PDC of lenacapavir was conservatively assumed to be equal to that of cabotegravir, but no less than 50%, as a single injection of lenacapavir provides coverage for an individual for 6 months of the 12‐month PDC estimate time frame. Populations with high PDC on cabotegravir may also benefit marginally from switching to lenacapavir. Therefore, in scenarios C1 and C2, PDC of lenacapavir was assumed to be equal to that of cabotegravir plus 5% (0.6 months) or 10% (1.2 months), respectively, based on the 7% (0.8 months) improvement in PDC from F/TDF to long‐acting cabotegravir (CAB‐LA), or fixed at 50%, whichever was greater. Real‐world PrEP effectiveness was defined as the product of PDC and intention‐to‐treat effectiveness, as reported from clinical trials and accounting for imperfect adherence (Figure 2A).

**TABLE 1:** Description of scenarios.

Uptake scenario	Description
S0	Baseline scenario; assumes 2025 levels of PrEP uptake for 2026–2030 without any additional uptake and without the introduction of lenacapavir.
S1	Increase in the number of PrEP users from 652,000 in 2025 to 1,150,000 in 2030. The percentage of F/TDF users remains stable over time. Moderate use of lenacapavir; 11% of PrEP users using lenacapavir in 2026 increasing to 37% by 2030 due to a combination of new initiations and individuals switching regimens.
S2	Increase in the number of PrEP users from 652,000 in 2025 to 1,150,000 in 2030. Lenacapavir is rapidly introduced; 13% of PrEP users using lenacapavir in 2026 increasing to 85% by 2030 due to a combination of new initiations and individuals switching regimens.
S3	Assumes 2025 levels of PrEP uptake for 2026–2030 without any additional uptake; 100% of PrEP users switch to lenacapavir.
S4	Increase in the number of PrEP users from 652,000 in 2025 to 1,150,000 in 2030; 100% of PrEP users switch to lenacapavir.
S5	Increase in the number of PrEP users from 652,000 in 2025 to 1,150,000 in 2030; no introduction of lenacapavir.
**PDC scenario**	
C0	PDC varies by age, race, gender, key population and insurance status^a^. Lenacapavir PDC set to 50% (6 months) or cabotegravir PDC, whichever is greater.
C1	PDC varies by age, race, gender, key population and insurance status^a^. Lenacapavir PDC set to 50% (6 months) or cabotegravir PDC^a^ + 5% (0.6 months), whichever is greater.
C2	PDC varies by age, race, gender, key population and insurance status^a^. Lenacapavir PDC set to 50% (6 months) or cabotegravir PDC^a^ + 10% (1.2 months), whichever is greater.

*Note*: Each simulation is a combination of one uptake scenario and one PDC scenario. The choice PDC scenario had no effect on lenacapavir‐free uptake scenarios S0 and S5.

Abbreviations: F/TDF, emtricitabine/tenofovir disoproxil fumarate; PDC, proportion of days covered; PrEP, pre‐exposure prophylaxis.

^a^ Cabotegravir PDC ranged from 28% to 81% (3.4–9.7 months); oral PrEP PDC ranged from 23% to 89% (2.8–10.7 months). See Table S3.

Six different PrEP uptake scenarios, labelled S0–S5, were considered based on general assumptions, including PrEP uptake, estimated number of PrEP indications, and recent trends in oral and injectable PrEP product choice, creating a potential range of possibilities that change as PrEP usage evolves through time (Table 1 and Figure 2). In the baseline scenario (S0), the estimated distribution of PrEP prescriptions between 2026 and 2030 was assumed to be the same as in 2025 (see Figure S1A), with no additional PrEP uptake and without the introduction of lenacapavir. In scenarios S1 and S2, the proportion of PrEP users is assumed to increase between 2026 and 2030. In S1, lenacapavir uptake is limited to the share of PrEP users prescribed cabotegravir or F/TAF in 2025, and in S2, lenacapavir gradually replaces all other PrEP options. In scenarios S3 and S4, it was assumed that all PrEP users will switch to lenacapavir starting in 2026, with overall PrEP use remaining steady in S3 and increasing in S4 at the same rate as in S1 and S2. Finally, in S5, it was assumed that overall PrEP use increases without the introduction of lenacapavir.

### 2.3 Incidence Model

To predict new primary HIV‐1 cases under each PrEP uptake scenario, a detailed model of HIV‐1 acquisition was developed (additional details in Supplementary Material, Table S5 and Figure S3). The number of new HIV‐1 cases in each stratum (county, race, age, gender, insurance status, injection drug use and sexual activity) was the product of weighted community prevalence, one minus the population‐level PrEP effectiveness and HIV‐1 transmission rate. The weighted community prevalence was calculated as the proportion of potential sexual and needle‐sharing partners with unsuppressed HIV‐1, using NHSS estimates of numbers of people living with HIV‐1 and rates of viral suppression (Figure S1B). The population‐level effectiveness of PrEP was defined as the reduction in HIV‐1 incidence, based on time‐varying PrEP coverage (Figure S4), PDC (Table S3) and clinical effectiveness derived from trial data (Table S4). The relationship between PrEP coverage and population‐level effectiveness within each stratum was weakly non‐linear to account for greater PrEP use by individuals with higher HIV exposure. Finally, HIV‐1 transmission rates accounted for differences owing to sexual activity, drug use, risk‐per‐act and population assortativity (Table S5). Note that in this model we use newly diagnosed HIV‐1 cases as a proxy for all HIV acquisitions and so do not account for changes in diagnostic rate over time.

To quantify uncertainty in PrEP effectiveness and HIV‐1 transmission rates, 1000 parameter sets were selected. To create a parameter set, clinical effectiveness was sampled from clinical trial data; the transmission parameters were then calibrated to HIV‐1 diagnosis data by county from 2022, the most recent year with complete data at the time, both overall and broken down by demographics [8]. Model calibration was performed in R version 4.4.0 with Markov Chain Monte Carlo (adaptMCMC 1.5). Full details on the incidence model are provided in the Supplementary Material and Table S6.

### 2.4 Measuring Intervention Impact

The number of new primary HIV‐1 cases under each combination of expansion scenarios S0–S5 and PDC scenarios C0–C2 were estimated for each year from 2026 to 2030. For scenarios S1–S5, the number of new HIV‐1 diagnoses averted was calculated as the difference in total HIV‐1 cases from 2026 to 2030 in the respective scenario and the baseline scenario S0.

### 2.5 Sensitivity Analyses

The primary analysis used diagnostic data from 2022, which may have been impacted by the COVID‐19 pandemic. Delays in diagnosis due to disruption in HIV‐1 services may have temporarily inflated new HIV‐1 diagnoses in subsequent years [26]. A sensitivity analysis was conducted by reducing HIV‐1 diagnoses by 10% across all scenarios to bring trends in line with pre‐COVID‐19 years (Figure S5).

In the primary analysis, PrEP uptake was higher among individuals with more exposure to HIV‐1, even within strata. Specifically, the calibrated model predicted that the first 50% of a population within a stratum to take PrEP would account for 66% (95% credible interval of 61%–71%) of potential new HIV‐1 cases. A sensitivity analysis was also conducted to simulate an “exposure‐neutral” distribution, in which 50% PrEP coverage within a population implied that 50% of potential new HIV‐1 cases were covered by PrEP.

### 2.6 Ethics and Consent

Ethical approval and informed consent were not required because this modelling analysis did not involve human subjects.

## 3 Results

### 3.1 Projected Change in New HIV‐1 Cases

**FIGURE 3:**
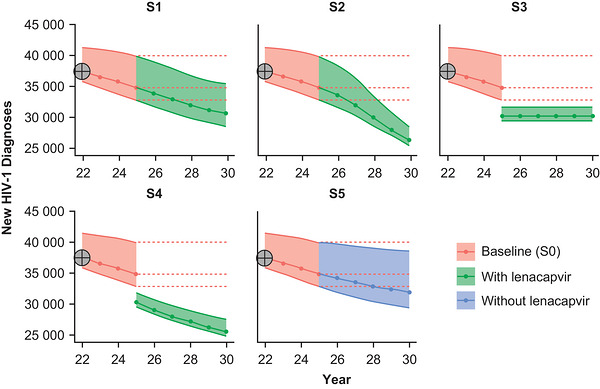
Number of new HIV diagnoses by year over time under different PrEP expansion scenarios. PDC scenario is C0. Red=baseline scenario (S0), green=scenarios with lenacapavir starting in 2026, blue=expansion of PrEP after 2025 with no lenacapavir. The model is calibrated to total acquisitions in 2022 (grey circle). The projected number of new HIV‐1 cases diagnosed annually was 34,800 (95% credible interval 32,800–39,900), indicated by the dashed horizontal lines. Abbreviations: PDC, proportion of days covered; PrEP, pre‐exposure prophylaxis.

With projected 2025 PrEP uptake (S0), it was estimated that 34,800 (95% credible interval 32,800–39,900) newly diagnosed HIV‐1 cases would occur (Figure 3). In S1, in which PrEP uptake increases and lenacapavir is gradually introduced (37% of PrEP users by 2030), annual HIV‐1 cases were projected to decrease by 12.2% (9.3%–15.2%) from 2025 to 2030 if the use of oral F/TDF remains high. In S2, in which PrEP uptake increases but lenacapavir expansion is more rapid (85% of PrEP users by 2030), new HIV‐1 cases were projected to decrease by 23.8% (19.7%–30.9%). In scenarios where all PrEP users switched to lenacapavir, cases were projected to decrease by 12.9% (7.4%–23.6%) if there was no increase in overall PrEP uptake (S3) and 26.2% (21.1%–35.2%) if there was PrEP expansion (S4). Finally, in S5, if PrEP uptake increased but lenacapavir was not introduced, HIV‐1 cases were projected to decline by only 8.4% (3.6%–10.8%) by 2030.

Scenarios with better PDC for lenacapavir (C1 and C2) resulted in greater reductions in HIV‐1 cases (Figure S6). Under scenario C2, in which lenacapavir PDC is improved by 10% compared with cabotegravir, switching all PrEP users to lenacapavir in 2026 without an increase in PrEP uptake (S3) reduced new HIV‐1 cases by 15.3% (12.1%–18.1%).

### 3.2 Projected Number of HIV‐1 Cases Averted

**FIGURE 4:**
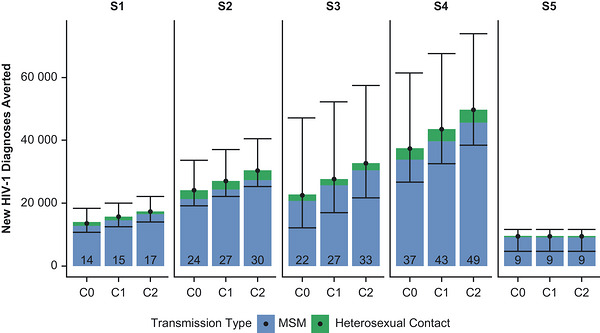
Newly diagnosed HIV‐1 cases averted between 2026 and 2030 in S1–S5 and C0–C2 in both MSM and heterosexual people. Uncertainty bars show 95% credible intervals for the overall total. Median number of new HIV‐1 cases averted (in thousands) is printed at the base of each bar. New HIV‐1 cases averted refers to a reduction in newly diagnosed HIV‐1 cases. The scenarios C0–C2 presented different assumptions about PDC for lenacapavir: C0, lenacapavir PDC set to 50% or cabotegravir PDC, whichever is greater; C1–C2, lenacapavir PDC set to 50% or cabotegravir PDC + 5% (C1) or 10% (C2), whichever is greater. Abbreviations: MSM, men who have sex with men; PDC, proportion of days covered.

In scenarios with an increase in PrEP uptake, moderate lenacapavir expansion (S1) averted 13,700 (10,800–18,300) cumulative new HIV‐1 cases between 2026 and 2030, while more rapid lenacapavir expansion (S2) averted 23,800 (19,100–33,700) new cases (Figure 4) over the same time frame. In S1 and S2, if lenacapavir PDC was increased by 10% (C2), 17,300 (14,000–21,900) and 30,200 (25,400–40,400) cases were averted, respectively. If all PrEP users switched to lenacapavir, 22,400 (12,200–47,200) new cases were averted when overall PrEP uptake did not increase between 2026 and 2030 (S3), and 37,400 (26,800–61,200) when overall PrEP uptake increased (S4). In S3 and S4, if lenacapavir PDC was increased by 10% (C2), 32,500 (21,700–57,200) and 49,400 (38,300–73,900) cases were averted, respectively. In S5, 9400 (4600–11,500) new HIV‐1 cases were averted when PrEP uptake increased, but lenacapavir was not introduced. The wide uncertainty in the estimates for S3 and S4 is driven by uncertainty in the effectiveness of oral PrEP and thus in the relative advantage of lenacapavir over oral PrEP.

### 3.3 Sensitivity Analyses

The number of new HIV‐1 cases averted in each scenario were robust to the sensitivity analyses (Figure S7). For S1 with PDC scenario C0, reducing overall new HIV‐1 cases to match pre‐COVID‐19 totals slightly reduced the estimated number of new HIV‐1 cases averted to 12,300 (9700–16,500). Using an exposure‐neutral PrEP distribution rather than an exposure‐informed distribution reduced the number of new HIV‐1 cases averted to 14,500 (11,600–17,600).

### 3.4 Subgroup Analyses

In PrEP uptake scenario S1 and PDC scenario C0, moderate lenacapavir expansion alongside an increase in the overall number of PrEP users averted 7.9% (6.0%–9.8%) of new HIV‐1 cases over a 5‐year span relative to S0. However, this reduction in HIV‐1 cases was not uniform across all populations with increased likelihood of acquiring HIV‐1. In all scenarios, it was assumed that PrEP use would remain negligible among people who inject drugs and significantly lower among heterosexuals than MSM. Consequently, the proportion of new HIV‐1 cases averted among MSM, 11.2% (8.2%–13.7%), was greater than that among heterosexual individuals, 2.3% (1.0%–3.1%) (Figure S8A). PrEP utilization was also much lower among those lacking insurance; lenacapavir averted 8.9% (6.7%–11.0%) of new HIV‐1 cases among those with any insurance compared with only 3.2% (2.2%–3.9%) among the uninsured (Figure S8B).

## 4 Discussion

In this modelling study, we demonstrated that twice‐yearly injectable lenacapavir has the potential to significantly improve the effectiveness of HIV‐1 prevention in the United States over the next 5 years. When lenacapavir use gradually replaced F/TAF and F/TDF use remained steady, 13,700 HIV‐1 cases were averted, an 8% reduction in cumulative new diagnoses compared with 2025 status quo PrEP use. This reduction in HIV‐1 cases varied across populations, with a greater proportion of new HIV‐1 cases averted in MSM (11%) compared with heterosexual individuals (2%). The projected number of cases averted increased when lenacapavir uptake was more rapid and became the primary PrEP option used in the United States. In scenarios where all PrEP users switched to lenacapavir, and PrEP use continued to expand, 37,400 HIV‐1 cases were averted over the subsequent 5 years. However, if PrEP use expanded but lenacapavir was not introduced, only 9400 new HIV‐1 cases were averted.

Our findings indicate that lenacapavir has the potential to markedly reduce the number of new HIV‐1 cases in the United States compared with what is achievable with the presently available PrEP options. Compared with daily oral PrEP, lenacapavir offers several important advantages, particularly in addressing adherence‐related challenges and expanding prevention options for individuals who may struggle with daily medication routines. In PURPOSE 1, conducted in cisgender women in sub‐Saharan Africa, F/TDF reduced HIV‐1 incidence by 29.9% compared with 100% for lenacapavir [5]. In PURPOSE 2, conducted among US MSM and gender‐diverse people, F/TDF reduced HIV‐1 incidence by 61% relative to background HIV‐1, compared with 96% for lenacapavir [4]. Prior model‐based projections of the impact of injectable cabotegravir have emphasized the importance of PrEP expansion rather than the mix of oral versus injectable products [20, 21]. These published models have assumed very high effectiveness for daily oral PrEP [20, 21]; therefore, improvements due to longer‐acting PrEP options are typically only marginal. However, Balasubramanian et al. noted that the increased PDC while using cabotegravir also plays a pivotal role in its impact on HIV‐1 incidence [19]. Lenacapavir will improve PDC intrinsically for many populations by providing at least 6 months of coverage. In all scenarios presented in the current analysis, a 10% increase in lenacapavir PDC compared to cabotegravir, similar to the PDC improvement from oral PrEP to injectable, further increased the projected number of HIV‐1 cases averted over 5 years by at least 20%.

The impact of PrEP can only be fully realized if it is widely available. Although legislation exists in the United States to reduce out‐of‐pocket expenses for PrEP prescriptions [27, 28], financial challenges still exist for many people who would benefit from PrEP [29, 30]. In this study, projected PrEP use, and consequently new HIV‐1 cases averted, was much lower among the uninsured population. Reduced funding and access to Medicaid may significantly undercut progress towards ending the HIV‐1 epidemic in the United States.

These predictions of averted new HIV‐1 cases have several limitations. First, a static cohort model was used rather than a dynamic model; therefore, only reductions in primary acquisitions were considered, and the model did not account for potential future demographic or behavioural shifts. Our estimates are, therefore, likely to be conservative as we do not account for the aversion of secondary transmission. Second, the timing of the COVID‐19 pandemic confounds a temporal analysis of concurrent changes in PrEP use and HIV‐1 incidence; therefore, the geographic correlation of PrEP use and HIV‐1 incidence in 2022 was used to calibrate the model. Third, these results are dependent upon the accuracy of the future PrEP scenarios, which are based upon current trends which can stall or even reverse [31]. For example, negligible PrEP use among people who inject drugs and relatively low PrEP uptake among heterosexuals was assumed; consequently, the relative reduction in HIV‐1 incidence in MSM was much greater than in heterosexual individuals. Fourth, our results are based on relatively conservative assumptions about the difference in effectiveness between oral and injectable PrEP, as (1) real‐world adherence to oral PrEP may be lower (or greater) than what is typical in a clinical trial setting and (2) lenacapavir (and cabotegravir) may continue to provide partial protection beyond 6 months (8 weeks) post‐injection. Fifth, PWID have extremely low PrEP uptake and adherence and so was assumed to be zero in this analysis [32]. The ongoing PURPOSE 4/HPTN 103 study will evaluate the pharmacokinetics, safety and acceptability of twice‐yearly lenacapavir in this population [33]. Modelling results are generally estimates and are dependent on the integration of model inputs, such as populations of interest. Clinicians, healthcare organizations and policy makers may adjust projected population numbers, proportions and other inputs to account for scenarios that are more representative of their specific patient population.

## 5 Conclusions

Expanding lenacapavir usage can significantly reduce HIV‐1 incidence and further progress towards ending the US HIV‐1 epidemic, even with the continued availability of oral PrEP. Twice‐yearly lenacapavir can improve adherence and time under protection, especially in populations that face barriers to PrEP access and uptake. However, the impact of lenacapavir is dependent on equitable access for both new and existing PrEP users.

## Author Contributions

Conceptualization: MM, DD, LM, SES, LT, JY, DM, JJ, JPCC, AH and WZ. Data curation: MM, SL and JY. Formal analysis: MM. Funding acquisition: LT and DD. Investigation: MM, DD and SL. Methodology: MM, DD, LM, SES, SL, LT, JY, DM, JJ and JPCC. Project administration: DD. Resources: DD. Software: MM. Supervision: DD. Validation: MM, SL, LT, JPCC and JJ. Visualization: MM, LT and JPCC. Writing of the original draft of the manuscript: MM and DD. Reviewing and editing the manuscript: All authors. All authors significantly contributed to the work reported, either in conception, study design, execution, acquisition of data, analysis and interpretation or in all these areas. All authors were involved in drafting, revising or critically reviewing the article for important intellectual content and gave final approval of the version to be published. MM had full access to all the data in the study and takes responsibility for the integrity of the data and the accuracy of the data analysis.

## Funding

The sponsor had a role in the design and conduct of the study in collaboration with the investigators. The sponsor had a role in the analysis and interpretation of the data; preparation, review or approval of the manuscript; and decision to submit the manuscript for publication. The sponsor did not control the decision regarding which journal was selected for submission.

## Conflicts of Interest

MM, LM, SL, SES and DD are supported by a research grant from Gilead Sciences, Inc. to Fred Hutch Cancer Center. LT, JY, DM, JJ, JPCC, AH and WZ are all employees and shareholders of Gilead Sciences, Inc.

## Supporting information



Supplementary Methods
Figure S1: (A) PrEP users per 100K (P_0, jt_ = 2025) in 2025 and (B) viraemia‐adjusted prevalence (vir.prev^0^) in each county.
Figure S2: Model‐predicted versus NHSS‐reported numbers of PrEP users per 100K by county and year, for counties with 12 or more PrEP users.
Figure S3: Observed versus predicted new HIV‐1 diagnoses per 100K stratified by transmission group and state.
Figure S4: Overall PrEP use in each county P_0, jt_ by year.
Figure S5: Sensitivity for number of new HIV‐1 diagnoses by year over time under different PrEP expansion scenarios.
Figure S6: Number of new HIV‐1 diagnoses by year over time under different PrEP expansion scenarios and PDC scenarios.
Figure S7: Sensitivity analysis for impact of COVID‐19 on new HIV‐1 cases averted in each scenario S1–S5 and C0–C2.
Figure S8: Relative proportion of new HIV‐1 cases averted in scenario S1 by (A) transmission group and (B) insurance status.
**Table S1**: Coefficients for the logistic regression model predicting proportion of injection drug users in each subpopulation.
**Table S2**: Number and rate of PrEP indications in the synthetic population.
**Table S3**: PrEP relative uptake (M), product choice (Q) and persistence (PDC).
**Table S4**: Marginal distribution of ITT effectiveness for each PrEP product.
**Table S5**: Assortativity parameters for HIV‐1 transmission.
**Table S6**: Transmission model parameters.

## Data Availability

Gilead Sciences, Inc. shares anonymized individual participant data upon request or as required by law or regulation with qualified external researchers based on submitted curriculum vitae and reflecting non‐conflict of interest. The request proposal must also include a statistician. Approval of such requests is at Gilead Science's discretion and is dependent on the nature of the request, the merit of the research proposed, the availability of the data and the intended use of the data. Data requests should be sent to datarequest@gilead.com.
