# Supplementary material for: Modelling the 5‐Year Impact of Twice‐Yearly Injectable Lenacapavir for HIV Pre‐Exposure Prophylaxis in the United States
Source: J Int AIDS Soc. 2026 Aug 10;29(8):e70188. doi: 10.1002/jia2.70188 (PMC13455683; doi:10.1002/jia2.70188)
Supplement: Supplementary file 1 [file JIA2-29-e70188-s001.docx]

**Supplementary Material**

**Supplemental Methods**

**Population structure**

A nested population structure was used with a total of 3,129 county or county equivalents (see **Figure 1**), indexed by $j$ and 294 subpopulations, indexed by $i$, subdividing each county by age (15–19, 20–24, 25–34, 35–44, 45–54, 55–64, or 65+ years), sex at birth, race (American Indian/Alaska Native, Native Hawaiian/Other Pacific Islander, Black, White, Hispanic, Asian, or multiple races), transmission group (men who have sex with men [MSM], heterosexuals, people who inject drugs [non-MSM], and MSM who inject drugs), and insurance status (any insurance and uninsured). The population size of each of these groups, $N_{ij}$, was estimated with the following steps:

1. US census estimates were used for the total population by county, race, age group, and sex at birth [1].
2. For insurance status (**Figure 1A**), census estimates were used for insurance status by age and race in each county. This data set used three age strata: < 19, 19–64, and 65+ years. The < 19 years’ insurance rate was applied to the 15–19 years population, the 19–64 rate to all populations 20–24 years through 55–64 years, and the 65+ rate to the 65+ years population.
3. The size of the MSM population (**Figure 1B**) was determined by the county-level frequency of individuals estimated to have had sex with men within 5 years [2]. It was assumed that MSM were proportionally distributed across all ages and racial groups.
4. The rate of injection drug users (**Figure 1C**) for subpopulation $i$ in county $j$, $P_{ij}^{\text{IDU}}$, was determined using the following formula:

$$\text{logit}\left( P_{ij}^{\text{IDU}} \right)=\beta_{0}+\beta_{\mathbf{X}}^{\text{IDU}}\mathbf{X}_{i}+\beta_{\text{SDOH}}^{\text{IDU}}\text{SDOH}_{j}+\beta_{\text{State}_{j}}^{\text{IDU}}$$

- by the demographics of the subpopulation, $\mathbf{X}_{i}$, social determinants of health ($\mathbf{SDOH}_{\boldsymbol{j}}$) and a state-level fixed effect. A two-stage calibration was used to estimate the regression coefficients $\beta_{0}$, $\beta_{\mathbf{X}}^{\text{IDU}}$, $\beta_{\text{SDOH}}^{\text{IDU}}$, and $\beta_{\text{State}_{j}}^{\text{IDU}}$. First, logistic regression was used to calibrate $\beta_{\text{SDOH}}^{\text{IDU}}$ and $\beta_{\text{State}_{j}}^{\text{IDU}}$ with data on county-level fatal drug overdoses from the national vital statistics system (NVSS) and SDOH data from the National HIV surveillance survey (NHSS) [3, 4]. Then Nelder-Mead optimisation was used to find the values of $\beta_{0}$ and $\beta_{\mathbf{X}}^{\text{IDU}}$ and match national-level estimates of injection drug users by age, race, and gender, and NHANES surveys on injection drug use among MSM (**Table S1**) [4-6].

For each subpopulation $i$, a pre-exposure prophylaxis (PrEP) indication rate was defined, $I_{i}$ (**Figure 1D**). Data from a national survey of MSM was used to assign rates of PrEP indication by age, keeping the rates constant by race and geography [7]. To estimate the number of people who inject drugs and heterosexual men and women with a PrEP indication, these estimates were reduced by a factor derived from CDC estimates of rates of PrEP indication (**Table S2**) [8].

**PrEP**

**PrEP uptake and persistence**

PrEP uptake, $P_{ijkt}$, was defined as the number of PrEP prescriptions per PrEP indication, in subgroup $i$, county $j$, and year $t$ and for product $k$. PrEP uptake was calculated from a county- and year-specific per-capita number of PrEP prescriptions $P_{0,jt}$ and a subgroup-specific PrEP multiplier, $M_{i}$, representing differences in PrEP uptake between populations. The proportion of people using different products varies by year and subgroup according to the variable $Q_{ikt}$ and the total number of PrEP users, regardless of product, is denoted $P_{ijt}$.

$$P_{ijkt}=\frac{Q_{ikt}}{\underset{k}{\sum}Q_{ikt}}\min\left[ \frac{P_{0,jt}M_{i}\underset{i}{\sum}N_{ij}}{\underset{i}{\sum}M_{i}I_{i}N_{ij}},1 \right]$$

$P_{0,jt}$ was calculated by county $j$ and year $t$ (**Figure S1A**), using a multi-level mixed effects model with state-, metropolitan statistical area-(MSA), and county-level intercepts and an MSA-level exponential growth rate using data from NHSS on PrEP users from 2012 to 2023. A national-level fixed effect was also included to describe the difference in growth from 2012 to 2017, and from 2017 onwards. County fits are summarised in **Figure S2**. In scenarios S0 and S3, $P_{0,j,t>=2025}^{\text{S0, S3}}=P_{0,j,t>=2025}$.

$M_{i}$ and $Q_{ikt}$ were calculated to match national-level trends in PrEP prescriptions by subgroups from 2021 to 2024. PrEP prescription data were obtained from the IQVIA Real-World Data Longitudinal Prescriptions and Pre-Adjudicated Claims (IQVIA LRxDx) Database. For each calendar year, PrEP uptake was quantified by the total number of individuals who received at least one PrEP prescription during that year. PrEP adherence was measured using the average annual proportion of days covered (PDC), calculated at the individual-level as the number of days covered by PrEP prescriptions during the year divided by the total number of days in the year, multiplied by 100. Both uptake and adherence measures were further stratified by key demographic and clinical subgroups, including age group, gender, race/ethnicity, PrEP regimen, and insurance type.

$$\begin{matrix} \log M_{i} & =\beta_{\text{Age}_{i}}^{\text{Uptake}}+\beta_{\text{Race}_{i}}^{\text{Uptake}}+\beta_{\text{Insurance}_{i}}^{\text{Uptake}}+\beta_{\text{MSM}_{i}}^{\text{Uptake}} \end{matrix}$$

When multiple PrEP products are available, the number of PrEP prescriptions for product $k$ was estimated using the following formula, calibrated to national-level PrEP prescription data from 2021 to 2024. Combining this model with PrEP uptake $P_{ijt}$ yields the current trend of PrEP uptake by year and product, $P_{ijkt}^{0}$.

$$\begin{matrix} \log Q_{ikt} & =\beta_{k,\text{MSM}_{i}}^{\text{Product}}+\beta_{k,\text{Insurance}_{i}}^{\text{Product}}+\beta_{k,\text{race}_{i}}^{\text{Product}}+\beta_{k,\text{age}_{i}}^{\text{Product}}\beta_{k,\text{Year}}^{\text{Product}}\left( t-2020 \right) \end{matrix}$$

Values for $Q_{ikt}$ for $t=2025$ are given in **Table S3**. Values for prior years can be found via $Q_{ikt}^{0}=Q_{ik,t=2025}\exp\left[ \beta_{k,\text{Year}}^{\text{Product}}\left( t-2025 \right) \right]$. For injectable PrEP $\beta_{\text{Injectables},\text{Year}}^{\text{Product}}$ = 0.972, for emtricitabine/tenofovir disoproxil fumarate (F/TDF) $\beta_{\text{TDF},\text{Year}}^{\text{Product}}$ = 0.044, for emtricitabine/tenofovir alafenamide (F/TAF) $\beta_{\text{TAF},\text{Year}}^{\text{Product}}$ = 0.035, and for mixed regimens $\beta_{\text{Mixed},\text{Year}}^{\text{Product}}$ = -0.120. The value of $Q_{ikt}$ for the years 2026–2030 varies by scenario. For S0 and S5, the choice of PrEP remains unchanged so $Q_{ik,t>2025}^{\text{S0, S5}}=Q_{ik,t=2025}^{0}$. For S1, $Q_{ik,t>2025}^{\text{S1}}=Q_{ik,t=2025}^{0}$ if $k=\text{F/TDF}$ or $k=\text{F/TDF and F/TAF mix}$. Otherwise,

$$\begin{matrix} Q_{ik,t>2025}^{\text{S1}} & =\frac{Q_{ikt}^{0}}{\underset{k'\in\{\text{F/TAF, CAB}\}}{\sum}Q_{ik',t=2025}^{0}} \end{matrix}$$

For S2, the trends follow $Q_{ikk}^{0}$ for all years. For S3 and S4, all PrEP is assumed to be lenacapavir; (i.e., $Q_{ik,t>2025}^{\text{S3, S4}}=1$ if $k=\text{lenacapavir}$ and $Q_{ik,t>2025}^{\text{S3, S4}}=0$ otherwise).

PrEP persistence was quantified by PDC, and the proportion of days for which individuals have study product available over the course of a year, starting from the day of PrEP initiation. PDC was described with a linear model. Values for $PDC_{ik}$ in population $i$ for product $k$ are shown in **Table S3**.

Three PrEP persistence scenarios for lenacapavir were considered.

1. C0: lenacapavir PDC is the same as for cabotegravir, due to both being long-acting products.
2. C1: lenacapavir PDC is increased by 5 percentage points across each population, to a max of 100%; this is similar to the PDC difference between cabotegravir and oral PrEP.
3. C2: lenacapavir PDC is increased by 10 percentage points across each population, to a max of 100%. As PDC for an individual receiving one dose of cabotegravir is 17% versus 50% for lenacapavir, a much greater PDC is possible; this was considered a more optimistic scenario.

**PrEP effectiveness**

Three forms of effectiveness were distinguished:

1. Intention-to-treat (ITT) effectiveness, $E_{ik}^{\text{ITT}}$, is the reduction in HIV incidence for individuals in population $i$ receiving a prescription for product $k,$ which was assumed to be comparable with an ITT effectiveness typically evaluated in a clinical trial. Effectiveness of F/TDF, F/TAF, and lenacapavir in heterosexual women is derived from the PURPOSE 1 study against a recency-assay-based counterfactual placebo. Similarly, effectiveness of F/TDF and lenacapavir in MSM was derived from the PURPOSE 2 study. Effectiveness of F/TAF and cabotegravir for MSM are derived from the DISCOVER and HPTN083 studies, respectively, using F/TDF as an active control. Cabotegravir efficacy was derived from HPTN 084 using a counterfactual placebo derived from external controls. Effectiveness for mixed regimens are taken to be the mean of the effectiveness for F/TDF and F/TAF. Due to the lack of data for heterosexual men and injection drug users, it was assumed that PrEP effectiveness in these populations is more akin to that of heterosexual women. A multivariate distribution was created for all clinical effectiveness parameter values by resampling the published HIV-1 incidence in each trial arm, counterfactual placebo arm, or, in the case of DISCOVER, incidence rate ratio using a log-normal distribution. As incidence was zero in the lenacapavir arm of PURPOSE 1, an exponential distribution was used. Values for the marginal distributions of $E_{ik}^{\text{ITT}}$ in MSM and heterosexual individuals are listed in **Table S4**.
2. Real-world effectiveness is the reduction in HIV-1 incidence for an individual over the course of a year after receiving a prescription. It is the product of clinical effectiveness and PDC, weighted by the proportion receiving each product $E_{i}^{\text{RWE}}=\underset{k}{\sum}E_{ik}^{\text{ITT}}\text{PDC}_{ik}Q_{ik}$.
3. Population effectiveness is the reduction in HIV-1 incidence for a population. It is dependent on real-world effectiveness, the total number of PrEP prescriptions, and the efficiency with which PrEP is prioritised towards those exposed to HIV-1.

$$\begin{matrix} E_{ijt}^{\text{Population}} & =f\left( P_{ijt} \right)E_{i}^{\text{RWE}} \\ f\left( P \right) & =\frac{\beta P}{\beta P+1-P} \end{matrix}$$

The function $f$ describes the relationship between coverage of individuals and coverage of HIV-1 exposure risk with $\beta=0$ representing risk-neutral PrEP distribution, and $\beta>0$ representing risk-informed PrEP distribution. The parameter $\beta$ was jointly fit with other transmission parameters, described below.

**HIV transmission**

HIV-1 incidence in population $i$, county $j$, and year $t$ ($H_{ijt}$) depends on 1) population-level PrEP effectiveness (defined above), 2) weighted community prevalence $\rho_{ij}$ which accounts for differences in HIV-1 prevalence and viral suppression by county, race, and transmission group, and 3) a transmission factor $\sigma_{i}$ which quantifies differences in HIV-1 transmission among different populations after accounting for differences in population prevalence.

$$\begin{matrix} H_{ijt} & =\sigma_{i}\left( 1-E_{ijt}^{\text{Population}} \right)\rho_{ij} \end{matrix}$$

**Weighted community prevalence**

$\rho_{ij}$ was defined as the prevalence of viremic HIV-1 among the pool of potential sexual or needle-sharing partners for population $i$ in county $j$. For each US county $j$, the following localised prevalence parameters were estimated using a multilevel, mixed-effects model incorporating county-, MSA-, and state-level random effects using data from [3]:

1. The overall diagnosed prevalence $\text{prev}_{j}^{0}$.
2. The diagnosed prevalence in each racial group $\text{prev}_{ij}^{1}$.
3. The diagnosed prevalence in each transmission group $\text{prev}_{ij.}^{2}$
4. The overall rate of viral suppression among diagnosed individuals $\text{vs}_{j}^{0}.$
5. The rate of viral suppression among diagnosed individuals in each racial group $\text{vs}_{ij}^{2}.$

From these, the prevalence of viremic HIV-1 was calculated at the county level ($\text{vir.prev}_{j}^{0}$), for each racial subgroup within each county ($\text{vir.prev}_{ij}^{1}$) and for each transmission group within each county ($\text{vir.prev}_{ij}^{2}$). It was assumed that 87% of people living with HIV-1 know their status ($\text{rate.diagnosis}=0.87$).

$$\begin{matrix} \text{vir.prev}_{j}^{0} & =\left( 1/\text{rate.diagnosis}-\text{vs}_{j}^{0} \right)\text{prev}_{j}^{0} \\ \text{vir.prev}_{j}^{1} & =\left( 1/\text{rate.diagnosis}-\text{vs}_{j}^{0} \right)\text{prev}_{ij}^{1} \\ \text{vir.prev}_{j}^{2} & =\left( 1/\text{rate.diagnosis}-\text{vs}_{ij}^{2} \right)\text{prev}_{ij}^{2} \end{matrix}$$

As the NHSS data lack information on viral suppression among different transmission groups, the county-level value was applied.

Weighted community prevalence, $\rho_{ijt}$, was defined as the prevalence of viremic HIV-1 in the pool of sexual or needle-sharing partners of subpopulation $i$. To calculate $\rho_{ijt}$ the following simplifying assumptions were made:

1. Assortative partnerships by race: Individuals select a proportion of partners, $\psi_{i}$, from the same racial or ethnic group, and the remainder from the population at large. This varies by key population, being lower among MSM (**Table S5**) [9].
2. Assortative partnerships by key population: Individuals select a proportion of partners, $\phi_{i}$, from the same key population and the remainder from the population at large. This proportion is assumed to be 100% for MSM and heterosexual men, but is lower for heterosexual women and injection drug users.
3. No intersectionality between transmission group and race: it was assumed that any transmission- or race-based differences in viremic prevalence from the population at large add linearly (on a log-odds scale).

$$\begin{matrix} g\left( \text{vir.prev}_{ij} \right) & =g\left( \text{vir.prev}_{ij}^{1} \right)+g\left( \text{vir.prev}_{ij}^{2} \right)-g\left( \text{vir.prev}_{j}^{0} \right) \\ g\left( x \right) & =\ln\left( x/\left( 1-x \right) \right) \\ \rho_{ij} & =\left( 1-\phi_{i} \right)\left( 1-\psi_{i} \right)\text{vir.prev}_{j}^{0} \\ & +\left( 1-\phi_{i} \right)\psi_{i}\left( 1-\text{vs}_{j}^{0} \right)\text{vir.prev}_{j}^{1} \\ & +\phi_{i}\left( 1-\psi_{i} \right)\left( 1-\text{vs}_{j}^{0} \right)\text{vir.prev}_{i}^{2} \\ & +\phi_{i}\psi_{i}\text{vir.prev}_{ij} \end{matrix}$$

**Model calibration, validation, and sensitivity analysis**

The joint distributions for clinical effectiveness $E_{ik}^{\text{clinical}}$, transmission multipliers $\sigma_{i}$, and risk-informed PrEP coverage parameter, $\beta$, were estimated via Markov Chain Monte Carlo (MCMC) (**Table S6**). For a single calibration, a total of 1,000 independent chains of length were ran, taking only the final step of each chain as a parameter sample. For each MCMC chain, $E_{ik}^{\text{clinical}}$ was sampled from the clinical-trial-based prior distribution which was fixed for the entirety of the chain, so that only $\sigma_{i}$ and $\beta$ were re-fit to the data.

The model was fit to county-level numbers of HIV-1 diagnoses in 2022. As a validation of the model, predictions were compared with state-level estimates, broken down by transmission group from 2017 and 2022 (see **Figure S3**). A sensitivity analysis was also performed in which the model was recalibrated with the constraint $\beta=0$.

**Supplemental Figures**

**Figure S1.** **A)** PrEP users per 100K ($P_{0,j,t=2025}$) in 2025 and **B)** Viremia-adjusted prevalence (vir.prev^0^) in each county.

Abbreviations: K, thousand; PrEP, pre-exposure prophylaxis.

**Figure S2.** Model-predicted versus NHSS-reported numbers of PrEP users per 100K by county and year, for counties with 12 or more PrEP users.

Black triangle indicates the national average across all counties with at least 12 PrEP users.
Abbreviations: K, thousand; NHSS, National HIV surveillance survey; PrEP, pre-exposure prophylaxis.

**Figure S3.** Observed versus predicted new HIV-1 diagnoses per 100K stratified by transmission group and state.

One-to-one line indicates a perfect agreement between model and data. The model was calibrated to county-level data from 2022 only, with results from 2017 included as a validation of model predictions. The size of each point reflects the absolute number of HIV-1 diagnoses in the population stratum.
Abbreviations: IDU, injection drug user; K, thousand; MSM, men who have sex with men.

**Figure S4.** Overall PrEP use in each county $P_{0,jt}$ by year.

$P_{0,jt}$ was defined as the number of PrEP prescriptions per capita.
Abbreviations: PrEP, pre-exposure prophylaxis.

**Figure S5.** Sensitivity for number of new HIV-1 diagnoses by year over time under different PrEP expansion scenarios.

PDC scenario is C0. Red=baseline scenario (S0), green=scenarios with lenacapavir starting in 2026, blue=expansion of PrEP after 2025 with no lenacapavir. Crosses indicate NHSS data for total number of new HIV-1 diagnoses. Model was calibrated to total HIV-1 infections in 2022 (grey circle) and then reduced by 10% to match pre-COVID-19 trends.
Abbreviations: NHSS, National HIV surveillance survey; PDC, proportion of days covered; PrEP, pre-exposure prophylaxis.

**Figure S6.** Number of new HIV-1 diagnoses by year over time under different PrEP expansion scenarios and PDC scenarios.

Red=baseline scenario (S0), green=scenarios with lenacapavir starting in 2026. Model was calibrated to total infections in 2022 (grey circle).
Abbreviations: PDC, proportion of days covered; PrEP, pre-exposure prophylaxis.

**Figure S7.** Sensitivity analysis for impact of COVID-19 on new HIV-1 cases averted in each scenario S1–S5 and C0–C2.

Uncertainty bars show 95% credible intervals for the overall total. Median number of HIV-1 infections averted (in thousands) is printed at the base of each bar. Infections averted refers to a reduction in newly-diagnosed HIV-1 cases.
Abbreviations: PrEP, pre-exposure prophylaxis.

**Figure S8.** Relative proportion of new HIV-1 cases averted in scenario S1 by **A**) Transmission group and **B**) Insurance status.

Numbers are relative to total infections predicted over a 5-year period with status quo 2025-level PrEP usage (S0). Infections averted refers to a reduction in newly diagnosed cases. ‘All’ category refers to entire population.
Abbreviations: MSM, men who have sex with men; PrEP, pre-exposure prophylaxis.

**Supplemental Tables**

**Table S1.** Coefficients for the logistic regression model predicting proportion of injection drug users in each subpopulation.

| Beta | Description | Coefficient |
| --- | --- | --- |
| $\beta_{0}$ | Intercept | 3.527 |
| $\beta_{X}$ | Male | 0 (reference) |
| $\beta_{X}$ | Female | -0.867 |
| $\beta_{X}$ | Age 18–39 years | 0 (reference) |
| $\beta_{X}$ | Age > 40 years | -0.521 |
| $\beta_{X}$ | Asian/other | 0 (reference) |
| $\beta_{X}$ | Black | 0.865 |
| $\beta_{X}$ | Hispanic | 1.017 |
| $\beta_{X}$ | White | 1.737 |
| $\beta_{X}$ | Non-MSM | 0 (reference) |
| $\beta_{X}$ | MSM | 0.893 |
| $\beta_{\text{SDOH}}$ | Poverty rate | 0.093 |
| $\beta_{\text{SDOH}}$ | Gini coefficient | 0.168 |
| $\beta_{\text{SDOH}}$ | Less than high school education | -0.002 |
| $\beta_{\text{SDOH}}$ | Median income | -0.877 |
| $\beta_{\text{SDOH}}$ | Severe housing burden | -0.078 |
| $\beta_{\text{SDOH}}$ | Urban | 0.088 |
| $\beta_{\text{SDOH}}$ | Uninsured rate | -0.118 |
| $\beta_{\text{SDOH}}$ | Unemployment rate | -0.057 |
| $\beta_{\text{SDOH}}$ | Syphilis rate | 0.153 |

For social determinants of health, rate values are all converted to log-odds. For counties with zero uninsured individuals or individuals facing a severe housing burden, one such individual was assumed. Log-transformed median income and log-transformed one plus the number of syphilis cases was used.
Abbreviations: MSM, men who have sex with men; SDOH, social determinants of health.

**Table S2.** Number and rate of PrEP indications in the synthetic population.

| Subgroup | Population (thousands) | PrEP indications (thousands) | Percent |
| --- | --- | --- | --- |
| Age 15–24 years | 43,846 | 541 | 1.23 |
| Age 25–34 years | 45,043 | 685 | 1.52 |
| Age 35–44 years | 43,232 | 752 | 1.74 |
| Age 45–54 years | 39,977 | 677 | 1.69 |
| Age 55–64 years | 41,566 | 544 | 1.31 |
| Age 65+ years | 57,128 | 239 | 0.42 |
| MSM | 4,996 | 1,409 | 28.19 |
| MSM/IDU | 233 | 66 | 28.50 |
| IDU (Male) | 2,568 | 543 | 21.16 |
| IDU (Female) | 1,155 | 238 | 20.65 |
| Heterosexual Contact (Male) | 125,591 | 284 | 0.23 |
| Heterosexual Contact (Female) | 136,250 | 898 | 0.66 |
| All | 270,793 | 3,438 | 1.27 |

Rates by age derived from Weiss et al., relative rates by key population derived from Smith et al [7, 8].
Abbreviations: IDU, injection drug user; MSM, men who have sex with men; PrEP, pre-exposure prophylaxis.

**Table S3.** PrEP relative uptake (M), product choice (Q), and persistence (PDC).

|  | | | | | Q: Product Choice (%) | | | | | PDC (%) | | | | |  |
| --- | --- | --- | --- | --- | --- | --- | --- | --- | --- | --- | --- | --- | --- | --- | --- |
| Age, years | Race | MSM | Ins. | M_i_ | | F/TAF | F/TDF | Mixed | CAB | | F/TAF | F/TDF | CAB | Mixed | |
| 15–24 | Asian/Other | N | Y | 1.0 | | 6.8 | 86.7 | 0.9 | 5.6 | | 34.2 | 31.0 | 38.1 | 46.0 | |
| 25–34 | Asian/Other | N | Y | 2.3 | | 7.5 | 82.5 | 1.3 | 8.7 | | 41.6 | 38.4 | 45.6 | 53.4 | |
| 35–44 | Asian/Other | N | Y | 1.5 | | 7.9 | 81.9 | 1.3 | 8.9 | | 47.3 | 44.1 | 51.2 | 59.1 | |
| 45–54 | Asian/Other | N | Y | 0.9 | | 9.0 | 81.4 | 1.4 | 8.2 | | 52.6 | 49.4 | 56.6 | 64.4 | |
| 55–64 | Asian/Other | N | Y | 0.7 | | 10.4 | 79.6 | 1.4 | 8.6 | | 50.9 | 47.7 | 54.9 | 62.7 | |
| 65+ | Asian/Other | N | Y | 0.6 | | 11.1 | 76.0 | 1.4 | 11.5 | | 49.6 | 46.4 | 53.5 | 61.4 | |
| 15–24 | Black | N | Y | 3.6 | | 7.2 | 79.6 | 0.9 | 12.3 | | 30.7 | 27.5 | 34.6 | 42.5 | |
| 25–34 | Black | N | Y | 8.2 | | 7.7 | 72.8 | 1.2 | 18.2 | | 38.1 | 34.9 | 42.1 | 50.0 | |
| 35–44 | Black | N | Y | 5.3 | | 8.1 | 72.0 | 1.2 | 18.7 | | 43.8 | 40.6 | 47.7 | 55.6 | |
| 45–54 | Black | N | Y | 3.4 | | 9.2 | 72.1 | 1.4 | 17.3 | | 49.1 | 45.9 | 53.1 | 60.9 | |
| 55–64 | Black | N | Y | 2.6 | | 10.6 | 70.1 | 1.4 | 17.9 | | 47.4 | 44.2 | 51.4 | 59.2 | |
| 65+ | Black | N | Y | 2.0 | | 11.0 | 64.5 | 1.3 | 23.2 | | 46.1 | 42.9 | 50.0 | 57.9 | |
| 15–24 | Hispanic | N | Y | 1.9 | | 8.9 | 80.1 | 1.1 | 9.9 | | 32.3 | 29.1 | 36.2 | 44.1 | |
| 25–34 | Hispanic | N | Y | 4.3 | | 9.5 | 74.1 | 1.5 | 14.9 | | 39.7 | 36.5 | 43.7 | 51.5 | |
| 35–44 | Hispanic | N | Y | 2.8 | | 10.0 | 73.3 | 1.5 | 15.3 | | 45.4 | 42.2 | 49.3 | 57.2 | |
| 45–54 | Hispanic | N | Y | 1.8 | | 11.3 | 73.0 | 1.6 | 14.0 | | 50.7 | 47.5 | 54.7 | 62.5 | |
| 55–64 | Hispanic | N | Y | 1.4 | | 13.0 | 70.8 | 1.6 | 14.6 | | 49.0 | 45.8 | 53.0 | 60.8 | |
| 65+ | Hispanic | N | Y | 1.0 | | 13.6 | 65.8 | 1.5 | 19.0 | | 47.7 | 44.5 | 51.6 | 59.5 | |
| 15–24 | White | N | Y | 3.8 | | 7.0 | 86.5 | 0.9 | 5.6 | | 35.3 | 32.1 | 39.2 | 47.1 | |
| 25–34 | White | N | Y | 8.8 | | 7.7 | 82.3 | 1.3 | 8.7 | | 42.7 | 39.5 | 46.7 | 54.5 | |
| 35–44 | White | N | Y | 5.7 | | 8.1 | 81.7 | 1.3 | 8.9 | | 48.4 | 45.2 | 52.3 | 60.2 | |
| 45–54 | White | N | Y | 3.7 | | 9.2 | 81.2 | 1.5 | 8.2 | | 53.7 | 50.5 | 57.7 | 65.5 | |
| 55–64 | White | N | Y | 2.8 | | 10.6 | 79.3 | 1.5 | 8.6 | | 52.0 | 48.8 | 56.0 | 63.8 | |
| 65+ | White | N | Y | 2.1 | | 11.4 | 75.7 | 1.4 | 11.5 | | 50.7 | 47.5 | 54.6 | 62.5 | |
| 15–24 | Asian/Other | Y | Y | 9.5 | | 31.2 | 63.6 | 2.5 | 2.7 | | 53.5 | 50.3 | 57.5 | 65.3 | |
| 25–34 | Asian/Other | Y | Y | 21.8 | | 33.6 | 58.9 | 3.5 | 4.0 | | 63.9 | 60.8 | 67.9 | 75.8 | |
| 35–44 | Asian/Other | Y | Y | 14.1 | | 34.7 | 57.7 | 3.4 | 4.1 | | 71.8 | 68.6 | 75.7 | 83.6 | |
| 45–54 | Asian/Other | Y | Y | 9.1 | | 37.8 | 54.9 | 3.7 | 3.6 | | 75.3 | 72.2 | 79.3 | 87.2 | |
| 55–64 | Asian/Other | Y | Y | 7.0 | | 41.8 | 51.2 | 3.5 | 3.6 | | 76.0 | 72.8 | 80.0 | 87.9 | |
| 65+ | Asian/Other | Y | Y | 5.2 | | 44.0 | 48.0 | 3.3 | 4.7 | | 71.6 | 68.4 | 75.5 | 83.4 | |
| 15–24 | Black | Y | Y | 34.0 | | 33.2 | 58.3 | 2.5 | 5.9 | | 50.0 | 46.9 | 54.0 | 61.9 | |
| 25–34 | Black | Y | Y | 77.7 | | 35.1 | 52.8 | 3.4 | 8.6 | | 60.4 | 57.2 | 64.4 | 72.2 | |
| 35–44 | Black | Y | Y | 50.5 | | 36.2 | 51.7 | 3.4 | 8.8 | | 68.3 | 65.1 | 72.2 | 80.1 | |
| 45–54 | Black | Y | Y | 32.4 | | 39.4 | 49.2 | 3.6 | 7.7 | | 71.8 | 68.7 | 75.8 | 83.7 | |
| 55–64 | Black | Y | Y | 24.8 | | 43.3 | 45.6 | 3.4 | 7.6 | | 72.5 | 69.4 | 76.5 | 84.4 | |
| 65+ | Black | Y | Y | 18.6 | | 44.9 | 42.1 | 3.2 | 9.9 | | 68.1 | 64.9 | 72.0 | 79.9 | |
| 15–24 | Hispanic | Y | Y | 17.9 | | 37.9 | 54.8 | 2.8 | 4.5 | | 51.6 | 48.4 | 55.6 | 63.4 | |
| 25–34 | Hispanic | Y | Y | 40.9 | | 40.0 | 49.6 | 3.8 | 6.5 | | 62.0 | 58.9 | 66.0 | 73.8 | |
| 35–44 | Hispanic | Y | Y | 26.5 | | 41.2 | 48.5 | 3.7 | 6.6 | | 69.9 | 66.7 | 73.8 | 81.7 | |
| 45–54 | Hispanic | Y | Y | 17.1 | | 44.5 | 45.8 | 3.9 | 5.8 | | 73.5 | 70.2 | 77.4 | 85.3 | |
| 55–64 | Hispanic | Y | Y | 13.1 | | 48.5 | 42.1 | 3.7 | 5.7 | | 74.2 | 71.0 | 78.1 | 86.0 | |
| 65+ | Hispanic | Y | Y | 9.8 | | 50.4 | 38.9 | 3.4 | 7.3 | | 69.7 | 66.5 | 73.6 | 81.5 | |
| 15–24 | White | Y | Y | 36.6 | | 31.7 | 63.0 | 2.6 | 2.7 | | 54.6 | 51.5 | 58.6 | 66.5 | |
| 25–34 | White | Y | Y | 83.6 | | 34.1 | 58.2 | 3.6 | 4.0 | | 65.0 | 61.9 | 69.0 | 76.9 | |
| 35–44 | White | Y | Y | 54.3 | | 35.3 | 57.1 | 3.5 | 4.1 | | 72.9 | 69.7 | 76.8 | 84.7 | |
| 45–54 | White | Y | Y | 34.9 | | 38.4 | 54.3 | 3.8 | 3.6 | | 76.5 | 73.3 | 80.4 | 88.3 | |
| 55–64 | White | Y | Y | 26.7 | | 42.4 | 50.5 | 3.6 | 3.6 | | 77.2 | 74.0 | 81.1 | 89.0 | |
| 65+ | White | Y | Y | 20.1 | | 44.6 | 47.3 | 3.4 | 4.7 | | 72.7 | 69.5 | 76.6 | 84.5 | |
| 15–24 | Asian/Other | N | N | 0.1 | | 4.8 | 84.0 | 1.0 | 10.2 | | 30.5 | 26.4 | 31.4 | 42.6 | |
| 25–34 | Asian/Other | N | N | 0.2 | | 5.2 | 78.1 | 1.3 | 15.3 | | 37.9 | 33.9 | 38.9 | 50.1 | |
| 35–44 | Asian/Other | N | N | 0.1 | | 5.5 | 77.5 | 1.3 | 15.7 | | 43.6 | 39.5 | 44.5 | 55.8 | |
| 45–54 | Asian/Other | N | N | 0.1 | | 6.3 | 77.7 | 1.5 | 14.6 | | 48.9 | 44.9 | 49.9 | 61.1 | |
| 55–64 | Asian/Other | N | N | 0.1 | | 7.3 | 76.0 | 1.5 | 15.2 | | 47.2 | 43.2 | 48.2 | 59.4 | |
| 65+ | Asian/Other | N | N | 0.0 | | 7.6 | 71.0 | 1.4 | 20.0 | | 45.9 | 41.8 | 46.9 | 58.1 | |
| 15–24 | Black | N | N | 0.3 | | 4.9 | 73.1 | 0.9 | 21.1 | | 27.0 | 22.9 | 27.9 | 39.1 | |
| 25–34 | Black | N | N | 0.7 | | 5.0 | 64.0 | 1.2 | 29.8 | | 34.4 | 30.4 | 35.4 | 46.6 | |
| 35–44 | Black | N | N | 0.4 | | 5.2 | 63.1 | 1.2 | 30.5 | | 40.1 | 36.0 | 41.1 | 52.3 | |
| 45–54 | Black | N | N | 0.3 | | 6.0 | 64.1 | 1.4 | 28.6 | | 45.4 | 41.4 | 46.4 | 57.6 | |
| 55–64 | Black | N | N | 0.2 | | 6.9 | 62.1 | 1.3 | 29.6 | | 43.7 | 39.7 | 44.7 | 55.9 | |
| 65+ | Black | N | N | 0.2 | | 6.9 | 55.0 | 1.2 | 36.9 | | 42.4 | 38.3 | 43.4 | 54.6 | |
| 15–24 | Hispanic | N | N | 0.1 | | 6.1 | 75.3 | 1.1 | 17.4 | | 28.6 | 24.5 | 29.5 | 40.8 | |
| 25–34 | Hispanic | N | N | 0.3 | | 6.3 | 67.0 | 1.5 | 25.1 | | 36.0 | 32.0 | 37.0 | 48.2 | |
| 35–44 | Hispanic | N | N | 0.2 | | 6.6 | 66.2 | 1.5 | 25.7 | | 41.7 | 37.6 | 42.6 | 53.9 | |
| 45–54 | Hispanic | N | N | 0.1 | | 7.6 | 66.8 | 1.7 | 23.9 | | 47.0 | 43.0 | 48.0 | 59.2 | |
| 55–64 | Hispanic | N | N | 0.1 | | 8.8 | 64.8 | 1.6 | 24.8 | | 45.3 | 41.3 | 46.3 | 57.5 | |
| 65+ | Hispanic | N | N | 0.1 | | 8.9 | 58.3 | 1.5 | 31.4 | | 44.0 | 39.9 | 45.0 | 56.2 | |
| 15–24 | White | N | N | 0.3 | | 5.0 | 83.9 | 1.0 | 10.2 | | 31.6 | 27.5 | 32.5 | 43.8 | |
| 25–34 | White | N | N | 0.7 | | 5.4 | 77.9 | 1.4 | 15.3 | | 39.0 | 35.0 | 40.0 | 51.2 | |
| 35–44 | White | N | N | 0.5 | | 5.6 | 77.3 | 1.4 | 15.7 | | 44.7 | 40.6 | 45.7 | 56.9 | |
| 45–54 | White | N | N | 0.3 | | 6.4 | 77.5 | 1.6 | 14.6 | | 50.0 | 46.0 | 51.0 | 62.2 | |
| 55–64 | White | N | N | 0.2 | | 7.5 | 75.8 | 1.6 | 15.2 | | 48.3 | 44.3 | 49.3 | 60.5 | |
| 65+ | White | N | N | 0.2 | | 7.8 | 70.8 | 1.5 | 20.0 | | 47.0 | 42.9 | 48.0 | 59.2 | |
| 15–24 | Asian/Other | Y | N | 0.8 | | 24.3 | 67.5 | 2.9 | 5.3 | | 49.8 | 45.8 | 50.8 | 62.0 | |
| 25–34 | Asian/Other | Y | N | 1.8 | | 26.0 | 62.0 | 4.1 | 8.0 | | 60.2 | 56.2 | 61.2 | 72.4 | |
| 35–44 | Asian/Other | Y | N | 1.1 | | 26.9 | 61.0 | 4.0 | 8.1 | | 68.1 | 64.0 | 69.1 | 80.3 | |
| 45–54 | Asian/Other | Y | N | 0.7 | | 29.7 | 58.8 | 4.3 | 7.2 | | 71.7 | 67.6 | 72.6 | 83.8 | |
| 55–64 | Asian/Other | Y | N | 0.6 | | 33.2 | 55.4 | 4.2 | 7.3 | | 72.3 | 68.3 | 73.3 | 84.5 | |
| 65+ | Asian/Other | Y | N | 0.4 | | 34.8 | 51.7 | 3.9 | 9.5 | | 67.9 | 63.8 | 68.8 | 80.1 | |
| 15–24 | Black | Y | N | 2.8 | | 25.3 | 60.4 | 2.9 | 11.4 | | 46.3 | 42.3 | 47.3 | 58.5 | |
| 25–34 | Black | Y | N | 6.3 | | 26.1 | 53.6 | 3.9 | 16.4 | | 56.8 | 52.7 | 57.7 | 68.9 | |
| 35–44 | Black | Y | N | 4.1 | | 27.0 | 52.5 | 3.8 | 16.6 | | 64.6 | 60.5 | 65.6 | 76.8 | |
| 45–54 | Black | Y | N | 2.6 | | 30.0 | 51.0 | 4.1 | 14.9 | | 68.2 | 64.1 | 69.1 | 80.3 | |
| 55–64 | Black | Y | N | 2.0 | | 33.3 | 47.8 | 4.0 | 14.9 | | 68.8 | 64.8 | 69.8 | 81.0 | |
| 65+ | Black | Y | N | 1.5 | | 34.0 | 43.4 | 3.6 | 19.0 | | 64.4 | 60.3 | 65.4 | 76.6 | |
| 15–24 | Hispanic | Y | N | 1.5 | | 29.6 | 58.3 | 3.3 | 8.8 | | 47.9 | 43.9 | 48.9 | 60.1 | |
| 25–34 | Hispanic | Y | N | 3.3 | | 30.8 | 52.0 | 4.4 | 12.8 | | 58.3 | 54.3 | 59.3 | 70.5 | |
| 35–44 | Hispanic | Y | N | 2.2 | | 31.8 | 50.9 | 4.3 | 12.9 | | 66.2 | 62.1 | 67.2 | 78.4 | |
| 45–54 | Hispanic | Y | N | 1.4 | | 34.9 | 48.9 | 4.6 | 11.5 | | 69.8 | 65.7 | 70.7 | 81.9 | |
| 55–64 | Hispanic | Y | N | 1.1 | | 38.6 | 45.6 | 4.4 | 11.4 | | 70.5 | 66.4 | 71.4 | 82.6 | |
| 65+ | Hispanic | Y | N | 0.8 | | 39.6 | 41.6 | 4.1 | 14.7 | | 66.0 | 61.9 | 67.0 | 78.2 | |
| 15–24 | White | Y | N | 3.0 | | 24.7 | 66.9 | 3.0 | 5.3 | | 51.0 | 46.9 | 51.9 | 63.1 | |
| 25–34 | White | Y | N | 6.8 | | 26.5 | 61.4 | 4.2 | 7.9 | | 61.4 | 57.3 | 62.3 | 73.5 | |
| 35–44 | White | Y | N | 4.4 | | 27.4 | 60.4 | 4.1 | 8.1 | | 69.2 | 65.2 | 70.2 | 81.4 | |
| 45–54 | White | Y | N | 2.8 | | 30.2 | 58.2 | 4.5 | 7.2 | | 72.8 | 68.7 | 73.7 | 85.0 | |
| 55–64 | White | Y | N | 2.2 | | 33.7 | 54.8 | 4.3 | 7.2 | | 73.5 | 69.4 | 74.4 | 85.7 | |
| 65+ | White | Y | N | 1.6 | | 35.4 | 51.1 | 4.0 | 9.4 | | 69.0 | 64.9 | 70.0 | 81.2 | |

Mixed was defined as a combination of F/TDF and F/TAF.
Abbreviations: CAB, cabotegravir; F/TAF, emtricitabine/tenofovir alafenamide; F/TDF, emtricitabine/tenofovir disoproxil fumarate; Ins, insurance; MSM, men who have sex with men; PDC, proportion of days covered; PrEP, pre-exposure prophylaxis.

**Table S4.** Marginal distribution of ITT effectiveness for each PrEP product.

|  | Median effectiveness (95% credible interval) | |
| --- | --- | --- |
| PrEP Product | MSM (including MSM/IDU) | Heterosexual contact/IDU |
| F/TAF only | 81.3 (40–94.3) | 16.3 (-27–45.4) |
| F/TDF only | 60.9 (18–81.2) | 30.1 (-21–60.1) |
| Cabotegravir only | 86.8 (66–94.8) | 94.6 (79–98.7) |
| Mixed regimen | 70.4 (33–86.7) | 22.4 (-15–48.0) |
| Lenacapavir only | 95.9 (83–98.9) | 98.2 (90–99.9) |

Mixed was defined as a combination of F/TDF and F/TAF. ITT effectiveness is the reduction in HIV-1 incidence for individuals prescribed each product, accounting for efficacy and adherence, but not PrEP coverage. Values are derived from the HPTN 083, HPTN 084, PURPOSE 1, PURPOSE 2, and DISCOVER studies. Note that the values in these tables are not independent as many products were tested concurrently against a common counterfactual (as in the PURPOSE and DISCOVER studies) or in active controlled studies (as in HPTN 083/4). For each model iteration, background HIV-1 incidence in the PURPOSE studies and in all trial arms was resampled using a log-normal distribution (or exponential distribution where the HIV-1 incidence was 0%) and PrEP ITT effectiveness was recalculated. CAB-LA effectiveness and F/TAF effectiveness in MSM were estimated by combining the HPTN 083/4 and DISCOVER studies with the effectiveness of F/TDF derived from the PURPOSE studies.
Abbreviations: CAB-LA, long-acting cabotegravir; F/TAF, emtricitabine/tenofovir alafenamide; F/TDF, emtricitabine/tenofovir disoproxil fumarate; IDU, injection drug user; ITT, intention-to-treat; MSM, men who have sex with men; PrEP, pre-exposure prophylaxis.

**Table S5.** Assortativity parameters for HIV-1 transmission.

| Population | $\psi$ | $\phi$ |
| --- | --- | --- |
| MSM | 0.35 | 1.0 |
| Injection drug users (non-MSM) | 0.70 | 0.9 |
| Heterosexual women | 0.70 | 0.9 |
| Heterosexual men | 0.70 | 1.0 |

Abbreviations: MSM, men who have sex with men.

**Table S6.** Transmission model parameters.

| Transmission parameter | Risk informed | Risk neutral |
| --- | --- | --- |
| Age 15–24 years | 1 (reference) | 1 (reference) |
| Age 25–34 years | 1.96 (1.89–2.04) | 1.95 (1.88–2.02) |
| Age 35–44 years | 1.29 (1.24–1.34) | 1.26 (1.21–1.32) |
| Age 45–54 years | 0.74 (0.70–0.78) | 0.73 (0.69–0.76) |
| Age 55–64 years | 0.49 (0.47–0.52) | 0.48 (0.46–0.51) |
| Age 65+ years | 0.13 (0.12–0.14) | 0.13 (0.12–0.14) |
| Male | 1 (reference) | 1 (reference) |
| Female | 1.35 (1.28–1.43) | 1.35 (1.28–1.42) |
| Heterosexual contact | 0.16 (0.15–0.18) | 0.18 (0.16–0.19) |
| MSM | 0.34 (0.30–0.37) | 0.35 (0.31–0.38) |
| MSM/IDU | 0.12 (0.11–0.13) | 0.13 (0.11–0.15) |
| IDU | 0.20 (0.18–0.22) | 0.22 (0.19–0.24) |
| American Indian/Alaska Native | 1 (reference) | 1 (reference) |
| Native Hawaiian/Other Pacific Islander | 0.75 (0.59–0.94) | 0.67 (0.53–0.85) |
| Black | 0.77 (0.70–0.86) | 0.71 (0.65–0.80) |
| White | 0.35 (0.32–0.39) | 0.32 (0.29–0.36) |
| Hispanic | 0.57 (0.52–0.64) | 0.53 (0.49–0.59) |
| Multiple races | 0.43 (0.38–0.49) | 0.40 (0.36–0.45) |
| Asian | 0.15 (0.14–0.18) | 0.14 (0.13–0.16) |
| Risk distribution (beta) | 1.98 (1.54–2.50) | 1.00 (1.00–1.00) |

Values represent medians with 95% credible intervals of 1,000 parameter sets estimated via MCMC.
Abbreviations: IDU, injection drug user; MCMC, Markov Chain Monte Carlo; MSM, men who have sex with men.

**References**

1.	Bureau USC. Census bureau data. 2024 [Available from: <https://data.census.gov>, including the population dataset located at <https://www2.census.gov/programs-surveys/popest/datasets/2020-2022/counties/asrh/cc-est2022-all.csv> and insurance rate datasets ACSDT1Y2023 C27001 and ACSDT5Y2023 C27001A, C27001B-I, C27002, and C27003 (accessed October 2025)].

2.	Grey JA, Bernstein KT, Sullivan PS, Purcell DW, Chesson HW, Gift TL, et al. Estimating the population sizes of men who have sex with men in US states and counties using data from the American Community Survey. JMIR Public Health Surveill. 2016;2(1):e14.

3.	CDC. National center for HIV, viral hepatitis, STD, and tuberculosis prevention. 2024 [Available from: <https://www.cdc.gov/nchhstp/index.html> accessed July 2025].

4.	CDC. NVSS: Drug Overdose Deaths. 2024 [Available from: <https://www.cdc.gov/nchs/nvss/drug-overdose-deaths.htm> accessed July 2025].

5.	Bradley H, Hall EW, Asher A, Furukawa NW, Jones CM, Shealey J, et al. Estimated number of people who inject drugs in the United States. Clin Infect Dis. 2023;76(1):96-102.

6.	CDC. Estimated percentages and characteristics of men who have sex with men and use injection drugs--United States, 1999-2011. MMWR Morb Mortal Wkly Rep. 2013;62(37):757-62.

7.	Weiss KM, Prasad P, Ramaraju R, Zlotorzynska M, Jenness SM. Estimated number of men who have sex with men with indications for HIV pre-exposure prophylaxis in a national sexual network study. J Acquir Immune Defic Syndr. 2020;84(1):10-7.

8.	Smith DK, Van Handel M, Wolitski RJ, Stryker JE, Hall HI, Prejean J, et al. Vital signs: estimated percentages and numbers of adults with indications for preexposure prophylaxis to prevent HIV acquisition--United States, 2015. J Miss State Med Assoc. 2015;56(12):364-71.

9.	Zang X, Krebs E, Min JE, Pandya A, Marshall BDL, Schackman BR, et al. Development and calibration of a dynamic HIV transmission model for 6 US cities. Med Decis Making. 2020;40(1):3-16.
